# ApoE‐ and Cfh‐deficient mice exhibit structural and molecular features of human early–intermediate retinal degeneration

**DOI:** 10.1002/ame2.70243

**Published:** 2026-07-01

**Authors:** Sergio Recalde, Maite Moreno Orduña, Jaione Bezunartea, Idoia Belza, Ainara Chas, Laura Fernández‐Sánchez, Nicolás Cuenca Navarro, Alfredo García‐Layana, Patricia Fernández‐Robredo, María Hernández

**Affiliations:** ^1^ Retinal Pathologies and New Therapies Group, Experimental Ophthalmology Laboratory, Department of Ophthalmology Clínica Universidad de Navarra Pamplona Spain; ^2^ Navarra Institute for Health Research, IdiSNA Pamplona Spain; ^3^ Department of Optics, Pharmacology and Anatomy University of Alicante Alicante Spain; ^4^ Department of Physiology, Genetics and Microbiology University of Alicante Alicante Spain

**Keywords:** aged macular degeneration, animal model, apolipoprotein E, Bruch's membrane, complement factor H, inflammation, lipid metabolism, retinal pigment epithelium

## Abstract

**Background:**

Age‐related macular degeneration (AMD) is a multifactorial retinal disease in which alterations in lipid metabolism and dysregulation of the complement system play a central role. The aim of this study was to characterize a novel double‐knockout (DK) mouse model deficient in apolipoprotein E and complement factor H (ApoE^−/–^Cfh^−/−^) as an experimental model of early and intermediate AMD.

**Methods:**

ApoE^−/–^Cfh^−/−^ mice and wild‐type controls underwent comprehensive morphological, ultrastructural, biochemical, and molecular analyses. Retinal and retinal pigment epithelium (RPE) integrity, Bruch's membrane (BM) morphology, lipid accumulation, complement activation, angiogenic signaling, and synaptic organization were evaluated using histology, electron microscopy, immunohistochemistry, biochemical assays, and gene expression analyses.

**Results:**

DK mice exhibited significant RPE thinning, disruption of tight junctions, vacuolization, and BM thickening (*p* < 0.05). Lipid accumulation and plasma lipid levels significantly increased compared with controls (*p* < 0.01). Complement activation was significantly enhanced, as evidenced by increased C5b‐9 deposition (*p* < 0.01). In addition, DK mice exhibited increased vascular endothelial growth factor expression (*p* < 0.05), altered matrix metalloproteinase activity (*p* < 0.05), and significant synaptic disorganization between photoreceptors and second‐order neurons (*p* < 0.05).

**Conclusions:**

The ApoE^−/–^Cfh^−/−^ mouse reproduces key molecular and structural features of early and intermediate retinal degeneration with statistically significant alterations. Although it does not progress to advanced disease stages, it represents a valuable model to investigate several factors of AMD pathogenesis and evaluate therapeutic strategies targeting early disease mechanisms.

## Graphical abstract

Age‐related macular degeneration (AMD) involves lipid dysregulation and complement overactivation. Here, we characterize a double‐knockout ApoE^−/−^/Cfh^−/−^ mouse as a model of early–intermediate retinal degeneration. These mice exhibit retinal pigment epithelium thinning, Bruch's membrane thickening, lipid accumulation, enhanced C5b‐9 deposition, increased vascular endothelial growth factor, altered matrix metalloproteinase activity, and synaptic disorganization. Together, these alterations reproduce several factors of early AMD features and intermediate retinal degeneration, making this model valuable for studying disease mechanisms and testing early‐stage therapeutic strategies.

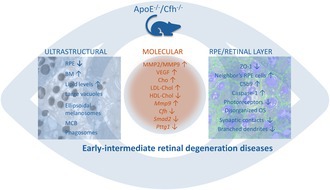

## 1 INTRODUCTION

Age‐related macular degeneration (AMD) represents a major cause of irreversible visual impairment and loss of quality of life in the elderly population worldwide.^1^, ^2^ The two main forms of AMD are dry (nonneovascular) and wet (neovascular). The advanced stage of dry AMD, known as geographic atrophy (GA), is characterized by progressive degeneration of the retinal pigment epithelium (RPE).^3^ Early stages of AMD are asymptomatic, whereas drusen—insoluble extracellular aggregates such as lipids, components of the complement cascade, and apolipoproteins^4^, ^5^—accumulate under the RPE,^6^ emerge abruptly, and rapidly progress to blindness.^7^, ^8^ Wet AMD is characterized by choroidal neovascularization (CNV) where new immature blood vessels grow from the choroid toward the outer retina. These vessels leak fluid below or within the retina leading to impaired vision,^9^ The etiology of AMD is complex and multifactorial, involving aging, genetic factors, and environmental or demographic risks (e.g., smoking, hypercholesterolemia).^10^, ^11^

A major genetic risk factor for AMD is a common variant in the complement factor H (CFH) gene (*Y402H*), which impairs the regulation of the complement system's alternative pathway. Supporting this link, CFH‐deficient mice (Cfh^−/−^) develop early AMD‐like features, including visual function decline and structural changes in the retina and Bruch's membrane (BM).^12^, ^13^, ^14^

Apolipoprotein E (*ApoE*) is involved in the transport of cholesterol and other lipids into cells through its ability to bind them^15^, ^16^ and is directly involved in the pathogenesis of AMD.^17^ The ApoE‐deficient mouse (ApoE^−/−^) has been extensively used in cardiovascular^18^, ^19^, ^20^, ^21^ and neurologic research^22^, ^23^, ^24^ as an animal model to study abnormal lipoprotein metabolism and hypercholesterolemia. Functional and morphological retinal changes have been observed in this animal model, suggesting it may provide valuable insight into the role of *ApoE* and cholesterol in retinal function.^25^, ^26^, ^27^

AMD primarily affects central vision and the macula; however, mice lack a macula or a comparable anatomical structure, and their immune system differs significantly from humans. Even so, many retinal‐degeneration genes first identified in mice have been linked to human disease. Moreover, several clinical, pathological, physiological, and biochemical features of AMD, such as retinal layer alterations, A2E (N‐retinylidene‐N‐retinylethanolamine) accumulation, abnormal ERGs (Electroretinograms), and RPE/photoreceptor degeneration, can be reproduced in mouse models.^25^, ^26^ Due to the need for animal models that recapitulate both early‐ and late AMD features, numerous genetically modified murine models have been produced. Ccl2^−/−^, Ccr2^−/−^, and ApoE^−/−^ mice exhibit retinal alterations, including photoreceptor and RPE atrophy or basal laminar deposits (BlamD) and vascular abnormalities in choriocapillaris (Ch).^25^, ^26^, ^27^, ^28^

To further elucidate the role of CFH deficiency and hypercholesterolemia in AMD, we produced double‐knockout (DK) mice with the genotype ApoE^−/−^/Cfh^−/−^. In these animals, retinal ultrastructure using transmission electron microscopy (TEM) and immunostaining were analyzed, RPE cell size and distribution were assessed, vascular endothelial growth factor (VEGF) protein expression and metalloproteinase activity were evaluated, gene expression of AMD‐related pathways was examined using real‐time polymerase chain reaction (RT‐PCR), and lipid profile was characterized.

## 2 MATERIALS AND METHODS

### 2.1 Animals

All experimental procedures were approved by the Animal Research Ethics Committee of the University of Navarra (no. 140‐12) and conducted according to the Association for Research in Vision and Ophthalmology Statement on the Use of Animals in Ophthalmic and Vision Research guidelines. The C57BL/6 mouse strain was the genetic background in current deficient animal models used in this study. Progenitor Cfh^−/−^ were supplied by Dr. Marina Botto (Imperial College, London), and ApoE^−/−^ were bought from Jackson Laboratories (002052, Bar Harbor, ME, USA). Plain knockout animals were crossed with the second generation of mice to obtain DK (ApoE^−/–^Cfh^−/−^) and control (wild type [WT], heterozygous for Cfh and ApoE) animals. These mice were then crossed, and the offspring were genotyped for Cfh and for ApoE using PCR. All the animals were euthanized at 12 months of age. The sample size varies for each methodology and is indicated in each figure legend.

### 2.2 Genotyping and experimental design

DNA for genotyping was extracted from ear tissue used for animal identification. The tissue was immersed in lysis buffer with proteinase K overnight at 56°C. The solution was centrifuged for 10 min at 10, 000 rpm, the supernatant was mixed with isopropanol, and the DNA strands were dissolved in water. PCR was carried out with primers for *ApoE* (ApoE‐F 5′‐GCCTAGCCGAGGGAGAGCCG‐3′, ApoE‐R 5′‐GCAGAGCCTTCGAAGCCAGC‐3′, and ApoE‐null 5′‐GCCG CCCCGACTGCATCT) and *Cfh* (Cfh‐F 5′‐GAAAATGGGTGGCGTCTAAC‐3′, Cfh‐R 5′‐ATTGAGATCCAACTGCCAGC‐3′, and Cfh‐null 5′‐GGATATGTCGGAGTAAGTAC‐3′), and the resulting DNA amplification bands were separated in 1% agarose gel for genotyping. The genome of several animals was confirmed using PCR sequencing by an external sequencing service (Applied Biosystems, Waltham, MA, USA).

### 2.3 Animal sacrifice, sample collection, and tissue homogenization for molecular purposes

The animals were euthanized with carbon dioxide. The eyes were enucleated and transferred to a saline solution (pH 7.4). Retinas were rapidly dissected by making a small incision with a scalpel 1 mm behind the limbus and extending the incision through 360° using fine ophthalmic scissors. Anterior segment structures (cornea, iris, and lens) were removed. RPE‐choroid (RPE‐Ch) samples were homogenized using a Teflon pestle in RIPA lysis buffer (Sigma‐Aldrich, St. Louis, MO, USA) and centrifuged for 20 min at 13 000 rpm at 4°C. The supernatant was collected, and total protein concentration was determined using the Bradford assay (Bio‐Rad, Hercules, CA, USA).

### 2.4 Transmission electron microscopy

The eyes from DK and WT mice were processed using conventional TEM.^29^ The samples were sectioned (1 μm thick) were using an ultramicrotome, stained with Toluidine blue O (198161, Sigma‐Aldrich), and examined under a light microscope to determine the areas of interest for measuring the RPE and BM. Thin sections (50–90 nm) were cut, collected on copper grids, and stained with 4% uranyl acetate and lead citrate. Subsequently, three sections from each animal were examined using a Zeiss EFTEM Libra 120 transmission electron microscope (Carl Zeiss, Thornwood, NY, USA), and images were obtained for later analysis. RPE and BM thicknesses were directly measured using ImageJ 1.44p (NIH, Bethesda, MD, USA) software in three different standardized locations in each image and averaged to provide a mean score for that micrograph. Quantification was performed by investigators blinded to the experiment. Genotype codes were revealed only after data analysis was completed.

### 2.5 Immunofluorescence of alterations in RPE and retinal layers

Eyes from animals in all experimental groups were enucleated and immediately fixed in 4% paraformaldehyde prepared in 0.1 M phosphate buffer (PB, pH 7.4) for 1 h. After fixation, the eyes were rinsed thrice in PB for 5 min each and cryoprotected in sucrose solutions (15% and 20% for 1 h each, followed by 30% for 24 h). The tissues were then embedded in OCT (optimal cutting temperature) compound (Tissue‐Tek, Sakura, Leiden, the Netherlands) and stored at −20°C. Retinal sections (12–14 μm) were obtained using a cryostat (Microm HM550, Thermo Fisher Scientific, Waltham, MA, USA) and preserved at −80°C until further analysis.

To examine changes in retinal layers, antibodies targeting proteins localized to distinct retinal cell types and synaptic contacts were employed (Table S1) to visualize photoreceptor cell structures: (i) mouse monoclonal anti‐rhodopsin, which labels rhodopsin (Rho) in rod outer segments (OS); (ii) mouse monoclonal anti‐bassoon, used to identify synaptic ribbons at the axon terminals of rod/cone photoreceptors; (iii) mouse monoclonal anti‐synaptophysin (SYN), which labels rod spherules and cone pedicles in the outer plexiform layer (OPL); and (iv) rabbit polyclonal anti‐γ‐transducin, used to identify cone photoreceptor cells. Interneurons within the inner nuclear layer (INL) were detected using (v) rabbit polyclonal anti‐calbindin (CB), which labels horizontal cells; and (vi) rabbit polyclonal anti‐protein kinase C‐α (PKC‐α), which marks ON‐rod bipolar cells. Finally, to assess apoptotic and inflammatory activities, (vii) rabbit monoclonal anti‐caspase‐1 and (viii) rabbit polyclonal anti‐C5b‐9 antibodies were used.

Retinal sections were washed in PB and incubated overnight with various combinations of primary antibodies diluted in PB containing 1% Triton X‐100. Sections were washed after 24 h in PB and incubated with the appropriate secondary antibodies (Alexa 555‐conjugated donkey anti‐mouse IgG, A31570, Invitrogen; Alexa 488‐conjugated donkey anti‐rabbit IgG, A21206, Invitrogen); DAPI (Sigma‐Aldrich) and TO‐PRO (1:1000; Molecular Probes) were used for nuclear labeling. Finally, sections were washed in PB and observed using confocal microscopy (Zeiss LSM800 and Leica TCS SP2). Specificity was confirmed using no‐primary‐antibody controls.^30^, ^31^

### 2.6 ZO‐1 immunofluorescence for RPE size and neighboring cell analysis

To study the morphological changes in tight junctions and size in RPE, we performed ZO‐1 immunofluorescence. After the animals were euthanized, their eyes were enucleated, and RPE‐Ch‐sclera complexes were fixed for 1 h in 4% paraformaldehyde/phosphate‐buffered saline (PBS) at 4°C. Samples were flat mounted and incubated with PBS, 3% Triton X‐100, 0.5% Tween 20.2% sodium azide, and 1% fetal bovine serum for 1 h at 4°C. Subsequently, the RPE‐Ch‐sclera complex was immunostained with anti‐mouse ZO‐1 (Zonula Occludens‐1) antibody (Table S1) overnight at room temperature. After 24 h the samples were incubated with donkey anti‐mouse 594 (Table S1). The areas were captured using confocal microscopy. Four images of the area near the optic nerve were obtained, and RPE cell size and the number of neighboring cells were measured automatically using a computer‐assisted color image analyzer developed by the Image Core Facility of the Center for Applied Medical Research (CIMA, Navarra). RPE cell segmentation was performed using a semiautomated algorithm (ImageJ with Trainable Weka Segmentation). Cells were classified into size categories (A–D) based on area quartiles derived from the WT distribution. The area of the RPE cells (μm^2^) is represented on a color scale between 100 and 1000 μm^2^ as follows: yellow white (100–315 μm^2^): type A cells, orange (315–530 μm^2^): type B cells, red (530–750 μm^2^); type C cells, and purple (750–1000 μm^2^): type D cells. Manual validation of segmentation accuracy was performed on 20% of randomly selected images by a second observer blinded to the experiment.

### 2.7 Lipid profile

Blood was collected with ethylenediaminetetraacetic acid and centrifuged for 10 min at 3000 rpm at 4°C to obtain plasma samples. Lipid profile, namely total cholesterol, high‐density lipoprotein cholesterol (HDL), and triglycerides, was analyzed using a Cobas Integra Analytical System C311 (Roche, UK).^32^

### 2.8 Gelatin zymography assay for matrix metalloproteinase activity measurement

Matrix metalloproteinase‐2 (MMP‐2) and MMP‐9 activity in RPE‐Ch homogenates was assessed using gelatin zymography. Supernatant samples (5 μg protein) were separated on 9% sodium dodecyl sulfate‐polyacrylamide gel electrophoresis gels containing 0.1% gelatin under nonreducing conditions. Gels were then washed, incubated in development buffer at 37°C for 18 h, fixed, stained, and analyzed using ImageQuant TL. The MMP activation ratio was defined as (active MMP)/(active + proMMP) band intensity.^33^

### 2.9 Western blot

VEGF protein expression on RPE‐Ch in WT and DK mice was measured using Western blot following the standard procedure.^34^ Five micrograms of total protein were analyzed using the primary antibody, monoclonal anti‐VEGF (0.2 μg/μL, sc7269, Santa Cruz Biotechnology Inc., Santa Cruz, CA, USA), at 4°C for 1 h, and the mixture was incubated with a horseradish peroxidase–conjugated goat anti‐mouse antibody (0.4 μg/μL, sc2005, Santa Cruz Biotechnology Inc.). Signals were detected using an enhanced chemoluminescence kit (ECLPrime WB detection kit, GE Healthcare, Fairfield, CT) and captured using ImageQuant 400 (GE Healthcare). The relative intensities of the immunoreactive bands were analyzed using ImageQuantTL software (GE Healthcare). Loading was verified using Ponceau S red, and the same blot was stripped and reblotted with an anti‐β‐actin monoclonal antibody (Sigma‐Aldrich) to normalize the VEGF levels.

### 2.10 Quantitative RT‐PCR

Total RNA was isolated from the mouse retina using ABI PRISM 6100 Nucleic Acid PrepStation (Life Technologies, Carlsbad, CA, USA). One thousand nanograms of each messenger RNA (mRNA) was reverse transcribed with the qScript cDNA Supermix kit (Quanta Biosciences Inc., Gaithersburg, MD, USA) using a 2720 Thermal Cycler (Life Technology). A total of 13 genes were analyzed based on their established roles in AMD‐relevant pathways: complement regulation (*Cfh*, Mm01299243; *Cfi*, Mm00432470), ECM remodeling (*Mmp2*, Mm00439498; *Mmp9*, Mm00442991; *endostatin*, Mm00487131; *Pai1*, Custom‐001), oxidative stress (*Sod1*, Mm01700393), apoptosis/pyroptosis (*caspase‐3*, Mm01195085), cell cycle (*Pttg1*, Mm00479224), aging/sirtuin pathway (*Sirtuin*, Mm00490758), transforming growth factor beta (TGF‐β) signaling (*Smad2*, Mm01262399), angiogenesis (*Vegf*, Mm01281449), and neuropeptide signaling (*Cck*, Mm00446170). For further statistical evaluations the level of applied housekeeping gene (*ACTβ*) was used as an endogenous control for data normalization. Relative quantification studies were performed from collected data (threshold cycle numbers, referred to as Ct) using 7300 System SDS, version 1.3 (Applied Biosystems). Relative quantity of the gene‐specific mRNA was calculated using DataAssist, version 2.0.

### 2.11 Statistical analysis

Data are presented as mean ± standard error of the mean. Statistical analysis was performed using analysis of variance (ANOVA) or the Kruskal–Wallis test, followed by Bonferroni or Mann–Whitney post hoc test, respectively. Statistical significance was accepted at the 95% confidence level (*p* < 0.05), and analysis was performed using SPSS (version 15.0, SPSS Inc., Chicago, USA).

## 3 RESULTS

### 3.1 Ultrastructural alterations of RPE and BM in DK mice

**FIGURE 1:**
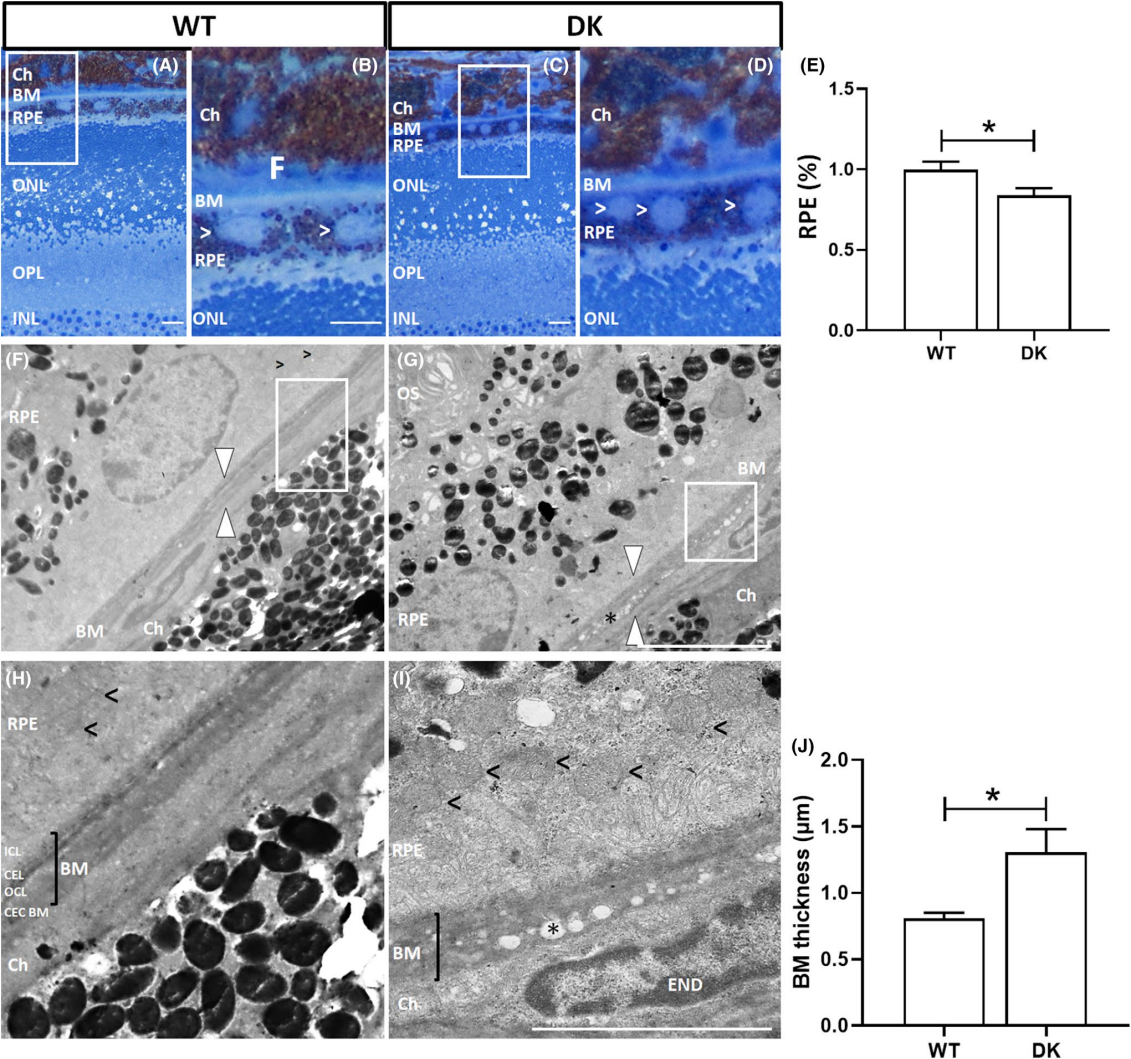
Changes in retinal pigment epithelium (RPE) and Bruch's membrane (BM) thickness in aged wild‐type (WT) and DK (double‐knockout) mice. (A, B) Semithin retinal section of WT mice labeled with Toluidine blue. (B) A magnification of the RPE area in the white square. (C, D) Semithin retinal section of DK mice labeled with Toluidine blue. (C) A magnification of the RPE area in the white square. Arrows in panels B and D indicate RPE nuclei. (E) Quantification of RPE thickness (%) in WT and DK mice. A significant decrease was observed in DK versus WT mice (*p* < 0.05). (F–I) Representative TEM images. (F, H) WT mice exhibit normal structure of RPE and Ch and normal thickness of BM (between white arrowheads) and several mitochondria in RPE (black arrowheads). (G, I) DK mice exhibit thickening of BM (between white arrowheads) with vacuolized structures (asterisks). White square indicates the magnified area analyzed. (J) Quantification of BM thickness (μm) in WT and DK mice with an increase in DK versus WT mice (*p < 0.05). *n* = 3–4 animals per group, 3 sections per animal, and 5–10 images per section. CEC BM, choroidal endothelial cell basement membrane; CEL, central elastic layer; Ch, choriocapillaris; ICL, inner collagenous layer; INL, inner nuclear layer; ONL, outer nuclear layer; OPL, outer plexiform layer; OS, outer segment; TEM, transmission electron microscopy. Scale bars: A–D, 50 μm; F–I, 10 μm.

DK mice exhibited several morphologic changes, mainly in the RPE and BM compared with WT mice. A decrease in RPE thickness in DK versus WT mice was observed from Toluidine blue–stained sections (Figure 1A–D). RPE nuclei were smaller in DK compared to WT mice (Figure 1B,D, arrows). This decrease showed statistically significant differences (0.99 ± 0.05 vs. 0.83 ± 0.04, *p* < 0.05) (Figure 1E). In TEM micrographs in DK mice, increased BM thickness (white arrows) was observed compared with the WT group (Figure 1F,G), and in magnified images (Figure 1H,I) we observed that the increase in BM thickness was often co‐localized with nonmembrane–bounded vacuolations (Figure 1G,I, asterisks) and surrounding intracellular organelles (Figure 1F,H, black arrows). This increase was statistically significant (0.807 ± 0.04 vs. 1.307 ± 0.17, *p* < 0.05) (Figure 1J).

**FIGURE 2:**
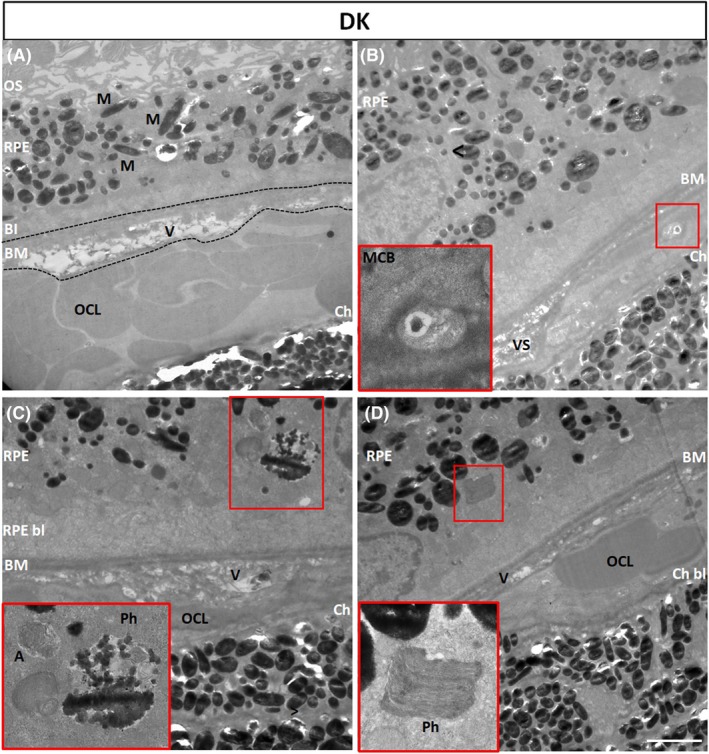
Ultrastructural alterations observed using transmission electron microscopy in DK (double‐knockout) mice. (A) Microimages show ellipsoidal melanosomes (M) in the RPE, with large vacuoles (V) in the BM. (B) Membranous cytoplasmic bodies (MCB) are present, with vacuoles close to the BM. Higher magnification of the MCB within the red square reveals that they are enclosed by a single membrane. (C) Higher magnification of the phagosomes (Ph) and autophagosomes (A) within the red square in the RPE shows accumulation of residual bodies: Enclosed by a single membrane in phagosomes and by a double membrane in autophagosomes. (D) Higher magnification of phagosomes in the RPE shows shed outer segment discs inside. *n* = 3–4 animals per group, 3 sections per animal, and 5–10 images per section. A, autophagosome; am, apical microvilli; BI, basal infolding; BM, Bruch's membrane; Ch, choriocapillaris; Ch bl, choriocapillaris basal lamina; n, nucleus; L, lipofuscin granules; Ph, phagosome; RPE, retinal pigment epithelium; RPE bl, RPE basal lamina. Scale bar: 10 μm.

At higher magnification of RPE‐BM‐Ch of DK mice, we observed ellipsoidal melanosomes (M) scattered throughout the apical region in RPE, and they appeared to be engulfed by host RPE cells (Figure 2A). Disrupted photoreceptor OSs are commonly observed over the RPE layer, where apical microvilli (am) enclose the distal tips of photoreceptor OSs (Figure 2A). Loss of continuity of the BM elastic layer (Figure 2A) with outer collagenous layer deposition and large vacuoles (V) and lipofuscin granules (L) were evident (Figure 2A,B) in DK mice. Disrupted architecture with membranous cytoplasmic bodies (MCB) (Figure 2B) was also detected between BM and Ch (Figure 2B, red square). Autophagosomes with double membranes (Figure 2C, red square) and phagosomes with a single membrane in RPE (Figure 2D, red square) containing shed OS discs were observed in DK animals.

### 3.2 RPE cell morphological changes in aged DK mice

**FIGURE 3:**
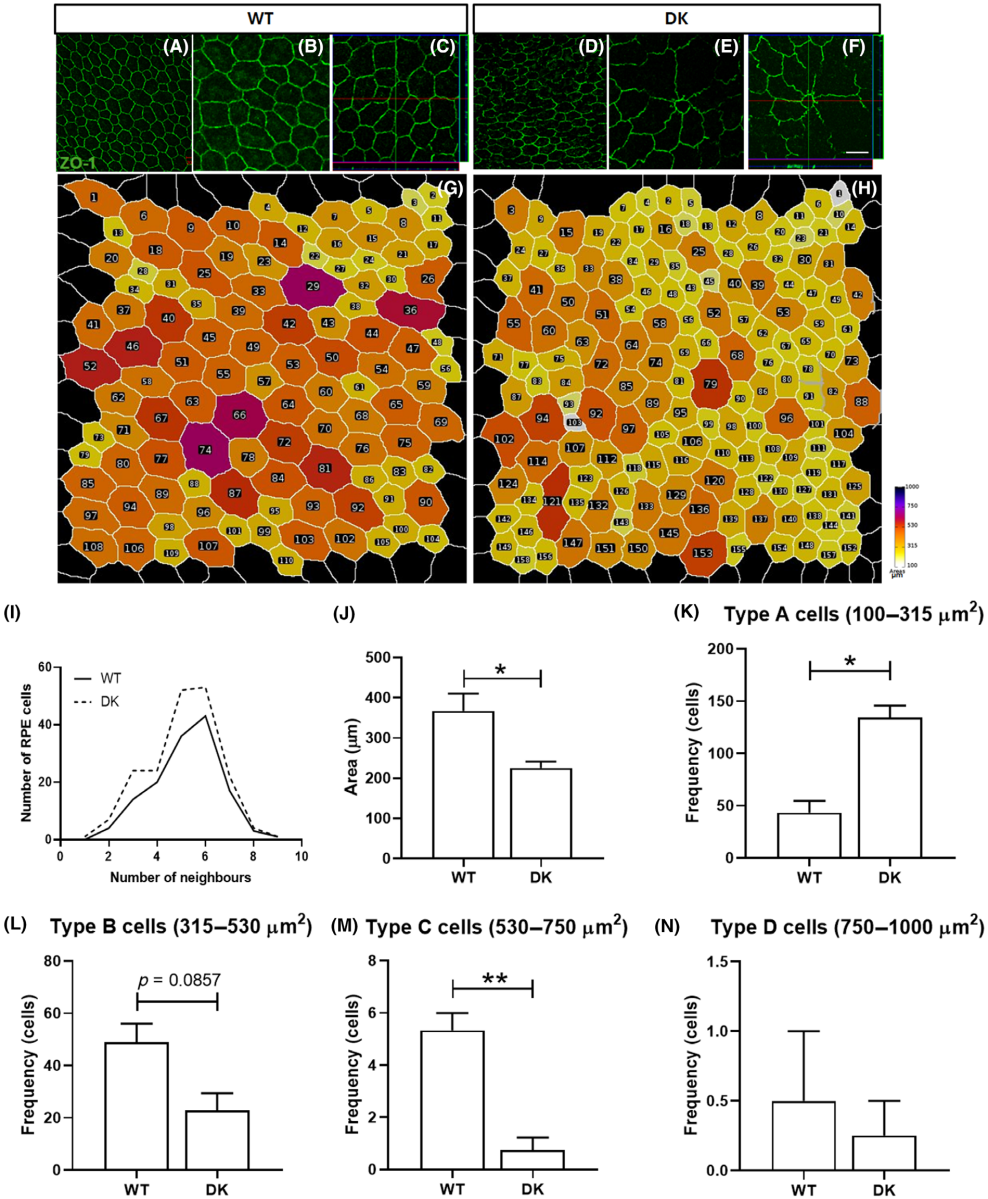
Analysis of RPE (retinal pigment epithelium) cell morphology, size, and number of neighbor cell changes in DK (double‐knockout) mice. (A–F) Representative confocal images of ZO‐1 immunofluorescence (green) in RPE flat mounts of (A–C) WT (wild‐type) and (D–F) DK mice show the tight junctions in RPE cells. Images B and E show higher magnification of the areas indicated in panels A and D. Images C and F show orthogonal projections of images B and E. (G, H) Representative mosaic of the RPE number, size, and neighbor cells in (G) WT and (H) DK mice. The area of the RPE cells (μm^2^) is represented in a color scale between 100 and 1000 μm^2^: Yellow white (100–315 μm^2^): Type A cells, orange (315–530 μm^2^): Type B cells, red (530–750 μm^2^): Type C cells, and purple (750–1000 μm^2^): Type D cells. (I) The number of RPE and neighbor cells shows an increase in DK mice, with more RPE cells surrounded by neighboring cells around them (maximum of up to 9). (J) Mean area (μm^2^) of the RPE cells in WT and DK mice shows a statistically significant decrease in RPE cell size in DK mice. (K–N) Frequency of (K) type A, (L) type B, (M) type C, and (N) type D in WT and DK mice shows a predominance of type A RPE cells in DK mice and a predominance of type C cells in WT mice. *n* = 4–7 animals per group; five images per section (*p < 0.05 and **p < 0.01). Scale bar: 50 μm.

DK mice stained with ZO‐1 (green) exhibited alterations of tight junctions between RPE cells compared with WT mice (Figure 3A–F). RPE cells are typically pentagonal or hexagonal shaped in WT mice (Figure 3A–C). In contrast, ZO‐1 immunolabeling showed a disordered pattern in DK mice with loss of protein from cell borders (Figure 3D–F). After quantification, the number of RPE cells in DK was significantly higher than that in WT mice (*p* < 0.05) (Figure 3G–I), and the area of RPE cells in DK was significantly smaller than that in WT mice (*p* < 0.05), as expected according to the semithin section observations (Figures 1C,D and 3J). Specific patterns were identified in relation to the distribution of large RPE cells. For instance, a group of large RPE cells, which we define as “neighbors,” was surrounded by a small, altered RPE cell (Figure 3H). In WT mice, the size and number of cells were homogeneous. The number of neighbor RPE cells increased in DK versus WT mice (Figure 3I). Cells were placed in groups A–D according to their size and frequencies calculated for each group. A predefined code color was assigned for the different areas as follows: yellow white (100–315 μm^2^): type A cells, orange (315–530 μm^2^): type B cells, red (530–750 μm^2^): type C cells, and purple (750–1000 μm^2^): type D cells. In Figure 3G,H WT mice exhibited a statistically significant frequency of large RPE cells (Figure 3G,H; color scale size: predominant red and purple) corresponding to RPE cell type C (*p* < 0.01) (Figure 3M), whereas in DK mice (color scale size: predominant orange and yellow), there was a statistically significant prevalence of RPE cell type A (*p* < 0.05) (Figure 3K). RPE cell type B was predominant in WT mice (*p* = 0.0857) (Figure 3L), and RPE cell type D was predominant in WT mice; however, there was no significant difference compared to DK mice (Figure 3N).

**FIGURE 4:**
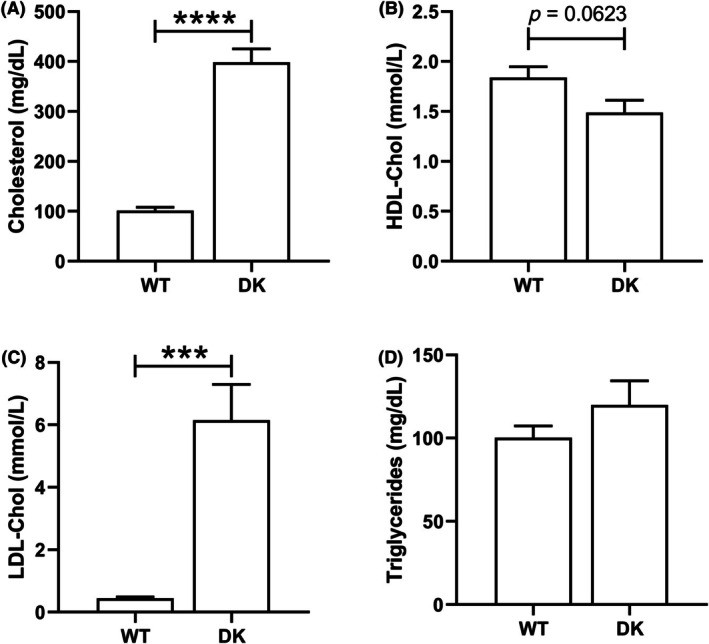
Lipid profile of WT (wild‐type) and DK (double‐knockout) mouse plasma. Plasma levels of (A) total cholesterol (Chol), (B) high‐density lipoprotein cholesterol (HDL‐Chol), (C) low‐density lipoprotein cholesterol (LDL‐Chol), and (D) triglycerides (TG) are shown. Chol was increased in DK versus WT mice (*p* < 0.0001), HDL‐Chol (*p* = 0.0623) and LDL‐Chol were significantly increased in DK versus WT mice (*p* < 0.001). TG levels were similar between groups. Chol and TG are expressed in mg/dL, and HDL and LDL‐Chol are expressed in nmol/L. *n* = 27 (17 WT and 10 DK animals). (***p < 0.001 and ****p < 0.0001).

### 3.3 Plasma lipid profile analysis using the Cobas Integra analytical system C311

Plasma cholesterol concentration was significantly higher in DK mice compared to WT mice (***p < 0.0001) (Figure 4A). High‐density lipoprotein cholesterol (HDL‐Chol) concentration tended to be higher in DK mice compared to WT mice, although this difference did not reach statistical significance (*p* = 0.0623) (Figure 4B), whereas a very significant increase was observed in low‐density lipoprotein cholesterol (LDL‐Chol) levels in DK mice compared to WT mice (***p < 0.001) (Figure 4C). Triglyceride concentration in plasma showed no significant differences between groups (Figure 4D).

### 3.4 MMP‐2 and MMP‐9 activity and VEGF expression

**FIGURE 5:**
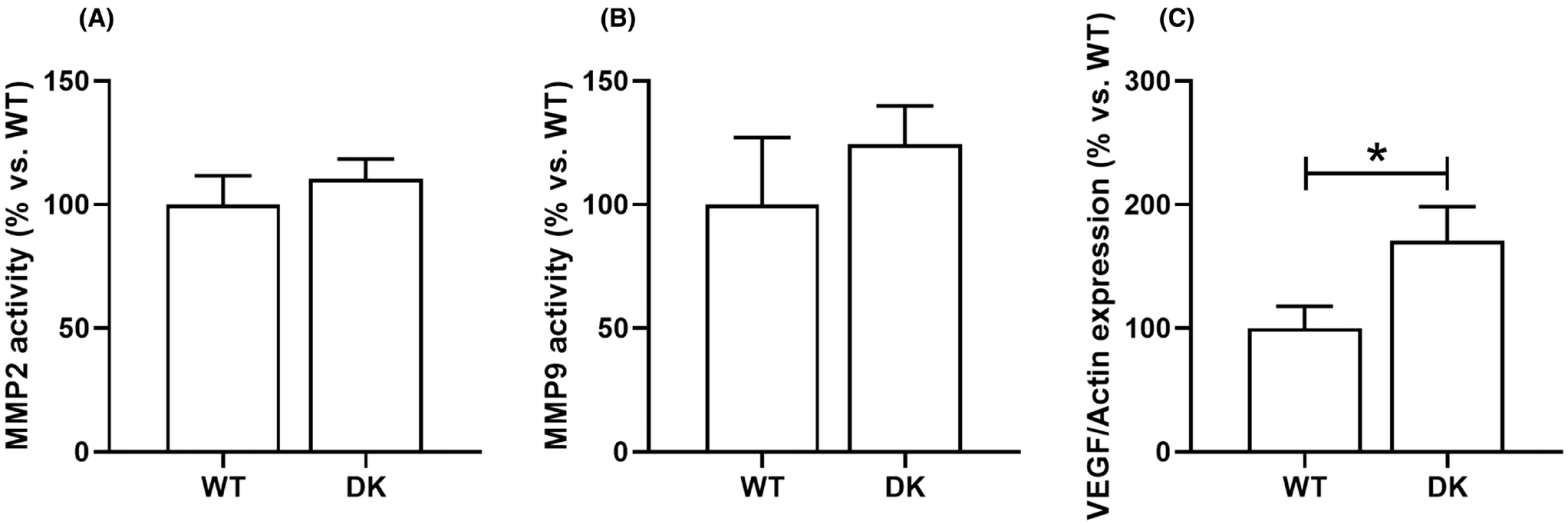
Gelatin zymography assay for matrix metalloproteinase‐2 (MMP‐2) and MMP‐9 activity and vascular endothelial growth factor (VEGF) protein expression in RPE (retinal pigment epithelium) homogenates from WT (wild type) and DK (double‐knockout) mice. (A, B) MMP activity was similar between WT and DK mice. (C) VEGF expression was significantly higher in DK than in WT mice. Representative MMP activity is shown in arbitrary units (AU) (total protein loaded: 5 μg; 5–6 eyes per group) (*p < 0.05). β‐Actin was blotted as an internal control. Data are presented as mean percentage ± SEM (standard error of the mean). *n* = 27 (17 WT and 10 DK animals).

Evaluation of MMP gelatinase activity in WT and DK mice indicated that MMP‐2 and MMP‐9 activity levels were higher in DK compared to WT animals; however, this difference was not statistically significant (Figure 5A,B). Moreover, there was a statistically significant activity increase (*p < 0.05) in VEGF protein expression in DK versus WT animals (Figure 5C).

### 3.5 Alteration in target genes of DK mice

**FIGURE 6:**
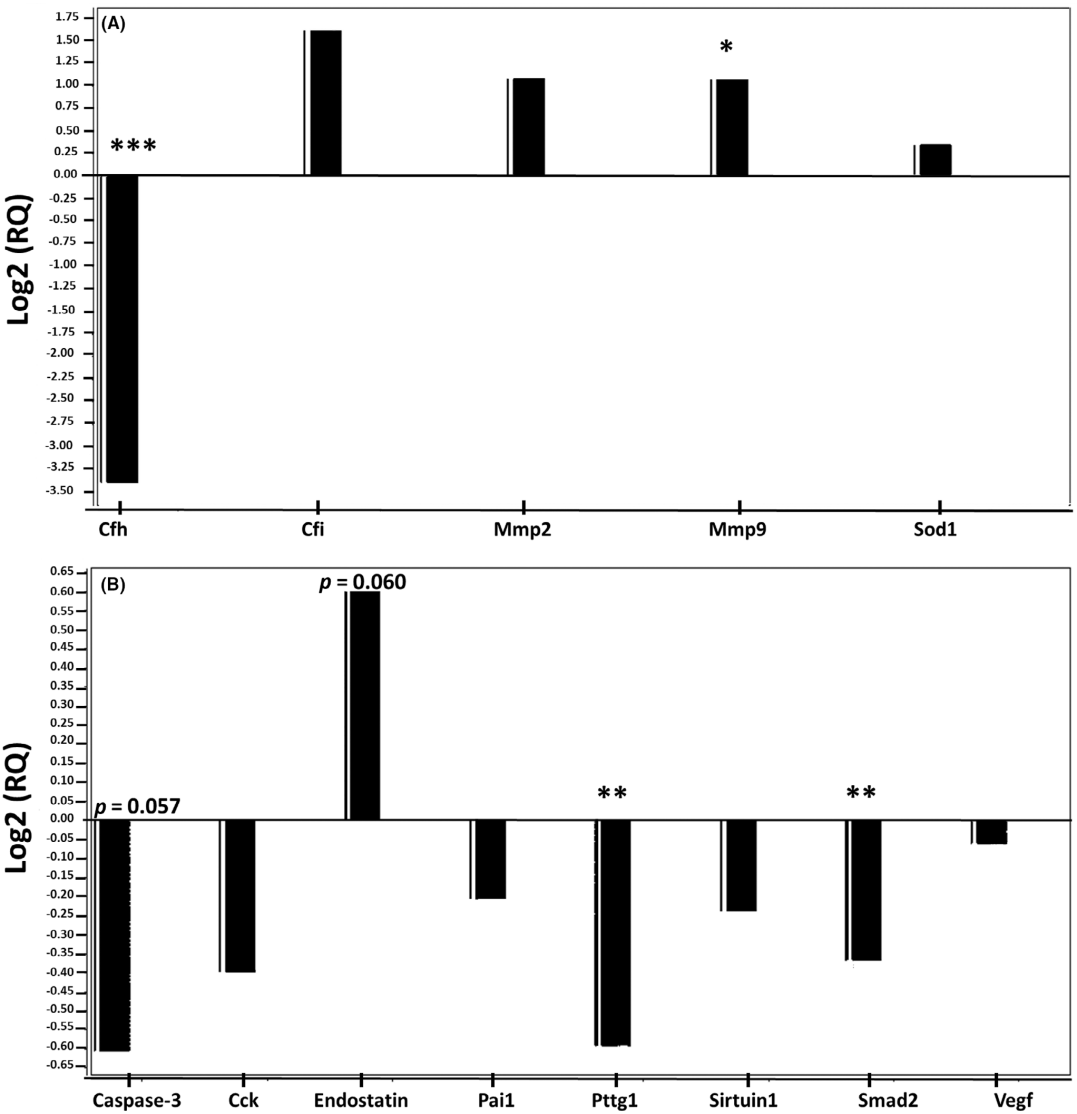
Quantitative analysis of a panel of target genes implicated in AMD (age‐related macular degeneration)–related pathways. (A) Expression levels of *Cfh*, *Cfi*, *Mmp2*, *Mmp9*, and *Sod1* are shown in DK (double‐knockout) retinas relative to WT (wild‐type) controls. (B) Expression levels of *caspase‐3*, *Cck*, *endostatin*, *Pai1*, *Pttg1*, *sirtuin*, *Smad2*, and *Vegf* are shown in DK mice relative to WT controls. Data are presented as mean fold change (DK/WT) ± SEM (standard error of the mean). *p < 0.05, **p < 0.01, and ***p < 0.001. *n* = 10 (5 WT and 5 DK animals).

We analyzed a panel of target genes involved in the AMD molecular pathway. Figure 6 shows the results obtained for the expression of *Cfh*, *Cfi*, *Mmp2*, *Mmp9*, *Sod1*, *caspase‐3*, *Cck*, *endostatin*, *Pai1*, *Pttg1*, *sirtuin, Smad2*, and *Vegf* genes in DK mice (Figure 6A,B). The results are presented as fold change in DK compared to WT animals. Downregulation of *Cfh* (*p* < 0.001), *Pttg1* (*p* < 0.01), and *Smad2* (*p* < 0.01) and upregulation of *Mmp9* (*p* < 0.05) were statistically significant in DK versus WT mice. *Caspase‐3* exhibited a trend toward downregulation (*p* = 0.057), and *endostatin* exhibited a trend toward upregulation (*p* = 0.060) in DK versus WT mice.

### 3.6 Caspase‐1 and C5b‐9 immunofluorescence

**FIGURE 7:**
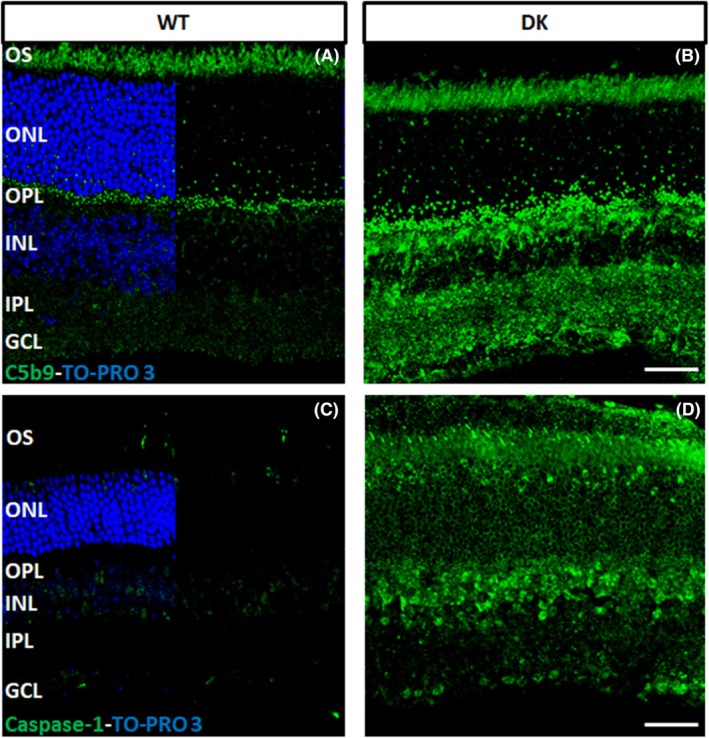
(A, B) C5b‐9 immunoreactivity (green) in representative retinal section from (A) WT (wild‐type) and (B) DK (double‐knockout) mice. There is an increase in C5b‐9 immunolabeling in the OPL (outer plexiform layer) and IPL (inner plexiform layer) in DK versus WT mice. (C, D) Caspase‐1 immunoreactivity (green) in retinal sections of (C) WT and (D) DK mice. Outer segments (OS), nuclei of photoreceptors, inner nuclear cells, and ganglion cells were positive to caspase‐1. *n* = 8 (3 WT and 5 DK animals). Cell nuclei were labeled with TO‐PRO‐3. Scale bar: 100 μm.

Local complement activation was evident in retinal sections of DK compared to WT mice by an increase in the deposition of the terminal complement activation product C5b‐9 (Figure 7A,B). Specifically, there was strong C5b‐9 staining in the OPL, inner plexiform layer (IPL), and ganglion cell layer (GCL) (Figure 7B). Moreover, caspase‐1 immunoreactivity intensity was higher in DK than in WT mice (Figure 7C,D). Caspase‐1 labeling was more intense in photoreceptor OSs and around the nuclei of photoreceptors, as well as in cells of the INL and GCL (Figure 7D).

### 3.7 Morphological changes in the retina of DK mice

**FIGURE 8:**
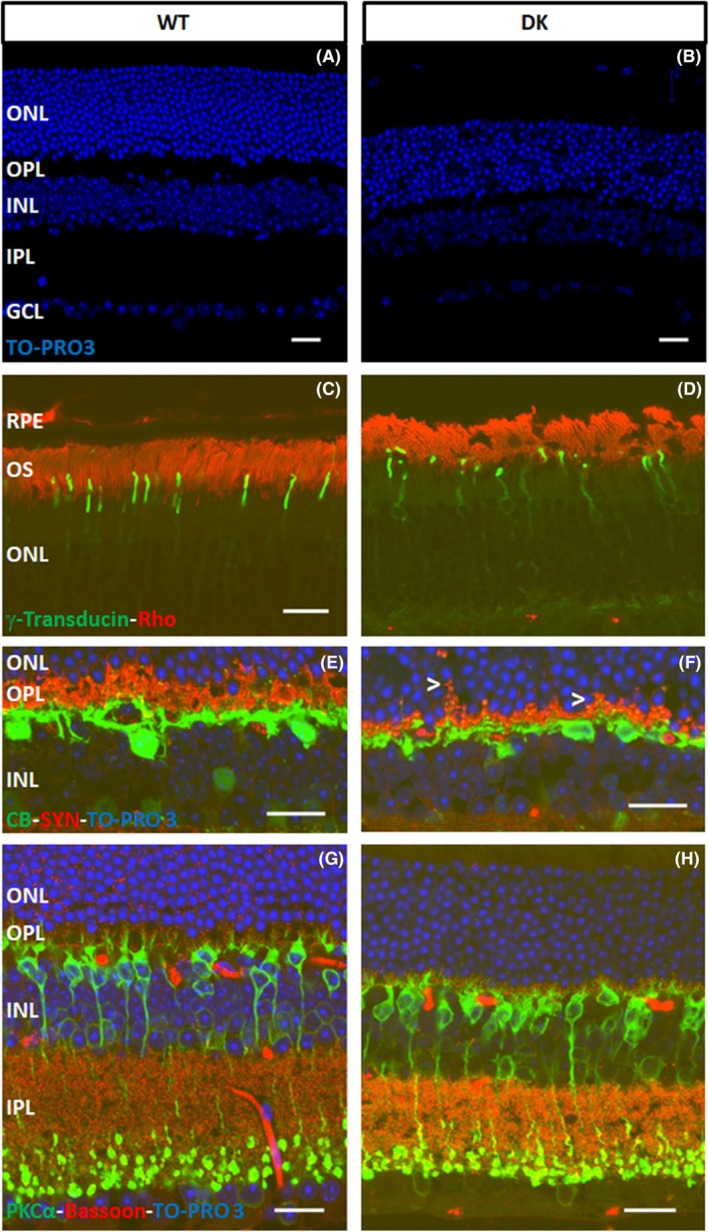
Retinal sections from (A, C, E, G) WT (wild type) and (B, D, F, H) DK (double‐knockout) mice immunostained with various specific markers. (A, B) Nuclear staining with TO‐PRO‐3 iodide (blue) shows a reduced thickness of the ONL and disorganization of photoreceptor rows in (B) DK mice compared with (A) control animals. (C, D) Immunolabeling of cone OS with γ‐transducin (green) and rod OSs with rhodopsin (red) reveals shorter and disorganized photoreceptor OSs in (D) DK mice relative to (C) WT mice. (E, F) Calbindin (CB, green), a marker for horizontal cells, and synaptophysin (red), a marker for photoreceptor axon terminals, show decreased synaptic contacts at the OPL in (F) DK mice compared to (E) WT mice. (G, H) Immunostaining with antibodies against PKC‐α (protein kinase C‐α, green) and bassoon (red) demonstrates normal dendritic arborization of rod bipolar cells in (G) WT mice, whereas (H) DK mice exhibit shorter and less dense dendrites. Additionally, fewer bassoon puncta can be observed in the OPL of DK mice compared with WT mice. INL, inner nuclear layer; IPL, inner plexiform layer; ONL, outer nuclear layer; OPL, outer plexiform layer; OS, outer segments. *n* = 8 (3 WT and 5 DK animals). Scale bar: 20 μm.

To investigate retinal morphology in DK mice, we performed immunohistochemical analyses using a panel of specific retinal markers. To assess the outer nuclear layer (ONL) thickness, TO‐PRO‐3 iodide was used as a nuclear stain. DK retinas exhibited a significant reduction in overall retinal thickness compared to WT (Figure 8A,B), accompanied by both thinning and disorganization of photoreceptor nuclei. In several regions, nuclei were absent at the ONL level. To further characterize photoreceptor morphology, double immunostaining was performed using antibodies against γ‐transducin (Figure 8C,D, green), a cone photoreceptor marker, and rhodopsin (Figure 8C,D, red), a rod OS marker. In WT retinas (Figure 8C), photoreceptor OS exhibited normal morphology, characterized by elongated and well‐organized parallel structures. In contrast, DK retinas (Figure 8D) exhibited shortened, frayed, and disorganized OS, with more pronounced alterations in cone OS.

Synaptic connectivity between photoreceptors and horizontal cells was examined using double immunolabeling for CB (Figure 8E,F, green), a horizontal cell marker, and SYN (Figure 8E,F, red), a presynaptic vesicle marker of photoreceptor axon terminals. At the OPL, DK retinas (Figure 8F) exhibited a reduction in synaptic contacts between photoreceptor terminals and horizontal cell dendrites compared to WT retinas (Figure 8E). In addition, some synaptic contacts appeared mislocalized within the ONL (Figure 8F, arrowhead).

To evaluate connectivity between photoreceptors and bipolar cells, immunostaining was conducted using PKC‐α (Figure 8G,H, green), a rod bipolar cell marker, together with bassoon (Figure 8G,H, red), a marker of synaptic ribbons at photoreceptor presynaptic terminals. In WT retinas (Figure 8G), rod bipolar cell dendrites exhibited extensive arborization with elongated dendritic processes. Conversely, DK retinas (Figure 8H) exhibited shorter and less branched dendrites, indicative of impaired synaptic connectivity. Furthermore, the number of bassoon‐immunoreactive puncta was reduced in DK retinas, suggesting a loss of synaptic contact between photoreceptors and bipolar cells.

## 4 DISCUSSION

This work is the first to present a DK mouse model (ApoE^−/−−^/Cfh^−/−^) that integrates two key pathways in AMD pathogenesis: complement regulation by Cfh, the principal gene linked to immune system and complement regulation; and lipid metabolism by ApoE, a polymorphic gene involved in lipid transport, essential for the catabolism of triglyceride‐rich lipoproteins at 12 months old, an age when structural and molecular changes are evident in mice. We demonstrate that the combined deletion of Cfh and ApoE produces a retinal phenotype, with multiple features associated with retinal degeneration similar to an early AMD in the RPE layer and an intermediate AMD in the outer and inner retinal layers.

Because AMD is inherently an age‐related and multifactorial disease, studying animal models at advanced ages is critical for capturing relevant pathological features. In both ApoE and CFH mouse models, structural and biochemical alterations in the retina, such as retinal thinning, vascular abnormalities, gliosis, drusen‐like deposits, and RPE dysfunction, become more pronounced or manifest only after 12 months old.^35^ Studies in ApoE4 knock‐in mice have demonstrated that retinal dysfunction and neuroinflammation are clearly evident at 52–57 weeks, whereas CFH knockout mice show progressive retinal changes and increased susceptibility to degeneration by 12 months. Younger animals, in contrast, often fail to exhibit the full spectrum of AMD‐like phenotypes.^36^, ^37^ Therefore, conducting studies in 12‐month‐old mice or older is essential to adequately model the progressive, age‐dependent nature of AMD and to ensure that experimental findings are translationally relevant to human disease.

The most significant structural outcome in DK mice is the early and progressive degeneration of the RPE‐BM complex, which compromises the blood–retinal barrier (Table 1). Key alterations include RPE atrophy characterized by significant RPE thinning, reduced nuclear size, and profound disruption of the typical hexagonal mosaic, with ZO‐1 mislocalization indicating loss of tight‐junction integrity. This pattern of RPE dedifferentiation and stress is consistent with observations in AMD patients and other complement‐dysregulated models.^38^, ^39^

**TABLE 1:** Summary of the results obtained in WT and DK mice.

	WT	DK
*Ultrastructure and morphology*		
RPE‐TEM	Normal RPE thickness and nuclei	*↓Thickness (smaller nuclei)
BM‐TEM	Normal BM thickness	*↑Thickness
Tight junctions (ZO‐1)	Normal RPE cell area (315–1000 μm^2^)	*↓RPE cell area (100–315 μm^2^)
RPE cells (ZO‐1)	Number of neighbor RPE cells between 1 and 8 (predominant 6 cells)	**↑**RPE cells with neighboring cells (predominant 4–6 cells)
*Protein*		
Chol	Normal level	****↑
HDL‐Chol	Normal level	**↓** (*p* = 0.0623)
LDL‐Chol	Normal level	***↑
Triglycerides	Normal level	NS
MMP‐2/MMP‐9	Normal level	NS
VEGF	Normal level	*↑
*Gene expression*		
*cfh*	Normal level	***↓
*mmp9*	Normal level	*↑
*caspase‐3*	Normal level	**↓** (*p* = 0.057)
*endostatin*	Normal level	**↑**(*p* = 0.060)
*pttg1*	Normal level	**↓
*Smad2*	Normal level	**↓
*Immunofluorescence in retinal sections*		
C5b9	OS and OPL	**↑** in OS, OPL, IPL, and GCL
Caspase‐1	Some OS and nuclei in INL	Strong labeling in OS, ONL, INL, and GCL
TO‐PRO‐3 (retinal nuclei cell)	10–12 photoreceptor rows	8–10 photoreceptor rows
γ‐Transducin	Cone OS	**↓**Cone OS
Rhodopsin	Rod IS	**↓**Rod IS
Calbindin	OPL and amacrine cells	**↓**IPL and small amacrine cells
Synaptophysin	Sprouting in OPL	**↓** In the sprouting in ONL
PKC‐α	Normal bipolar cells	Altered bipolar cells, axons, and nuclei
Bassoon	OPL and IPL	**↑** In OPL and **↓** in thickness

Abbreviations: BM, Bruch's membrane; DK, double knockout; GCL, ganglion cell layer; HDL, high‐density lipoprotein; INL, inner nuclear layer; IPL, inner plexiform layer; IS, inner segment; LDL, low‐density lipoprotein; MMP, matrix metalloproteinase‐2; ONL, outer nuclear layer; OPL, outer plexiform layer; OS, outer segment; PKC‐α, protein kinase C‐α; NS, no significance; RPE, retinal pigment epithelium; TEM, transmission electron microscopy; VEGF, vascular endothelial growth factor; WT, wild type. *p < 0.05, **p < 0.01, ***p < 0.001 and ****p < 0.0001.

DK mice showed a BM remodeling exhibiting a pronounced thickening, nonmembrane‐bound vacuolization, disrupted elastic layers, changes in the RPE basal lamina, and accumulation of lipofuscin and MCBs. Thickening of the RPE basal lamina closely resembles RPE BlamD deposits, a hallmark of AMD^40^; however, additional techniques such as OCT would be necessary to confirm our findings. Furthermore, these abnormalities are more consistent with early ECM remodeling described in aged human AMD and ApoE^−/−^ animal models.^41^, ^42^

The presence of autophagosomes and phagosomes observed in DK mice is consistent with possible impairments in autophagy and OS degradation, though mechanistic studies are required, consistent with RPE dysfunction and oxidative stress, two well‐established mechanisms in AMD progression.^43^ While extensive lipofuscin accumulation was modest in these DK mice, consistent with an early–intermediate AMD phenotype, the observed BM pathology aligns with the “garbage can” hypothesis of aging BM, where lipid and protein waste accumulation drive dysfunction.^44^

The DK phenotype is underpinned by synergistic systemic and molecular dysregulation with dyslipidemia and complement dysregulation. DK mice exhibited elevated total and low‐density lipoprotein cholesterol with a trend toward reduced HDL, a profile characteristic of ApoE^−/−^ mice that exacerbates BM lipid deposition and RPE metabolic stress.^29^, ^45^ As expected from Cfh deletion, DK retinas exhibited strong C5b‐9 deposition across all retinal layers, confirming uncontrolled complement activation. This mirrors the complement dysregulation seen in AMD patients with CFH risk alleles and in Cfh^−/−^ mice.^46^, ^47^, ^48^, ^49^

A pro‐inflammatory molecular profile was evident, with overexpression of *Mmp9* (a gene linked to exudative AMD) and an increasing trend in MMP activity that was not statistically significant, suggesting enhanced ECM turnover contributing to BM pathology. Concurrently, elevated VEGF protein and increased caspase‐1 immunoreactivity suggested a possible inflammasome pathway engagement, indicating a pro‐angiogenic and pro‐inflammatory environment linking chronic inflammation to potential neovascular progression.^50^, ^51^

The increase in VEGF protein had already been reported in other studies by the group using the CNV mouse model.^30^, ^33^ However, in DK animals, the expression of *endostatin*, an anti‐angiogenic factor, was increased. Similar results have been observed in other pathologies, such as hepatocellular carcinoma.^52^ In that study, both VEGF protein (pro‐angiogenic) and *endostatin/collagen XVIII* gene expression (anti‐angiogenic) were elevated in patients, and interestingly, there was a positive correlation between the two. This is not a contradiction but rather a compensatory response triggered by uncontrolled angiogenic stimulation.

The structural moderate defects in the RPE‐BM complex culminate in significant photoreceptor and synaptic pathology. DK mice exhibit thinning of the ONL and photoreceptor disruption, which, although more severe than typical early human AMD, may reflect accelerated pathology due to complete gene deficiency. Synaptic analyses revealed reduced connectivity between photoreceptors and second‐order neurons (bipolar and horizontal cells), along with mislocalized synaptic contacts and decreased bassoon‐positive puncta. This synaptic disassembly likely underlies the functional deficits (altered scotopic a‐wave) reported in aged Cfh^−/−^ mice and provides a morphological correlate for vision loss in AMD.^14^ The synaptic and photoreceptor changes may reflect a “murine‐specific response” to chronic RPE stress and complement activation, and that extrapolation to human disease stages requires caution.

The DK model integrates and amplifies features reported in the literature. Similar to ApoE^−/−^ mice, DK mice exhibit BM vacuolization and thickening,^41^ and similar to Cfh^−/−^ mice they exhibit complement activation as occurs in Cfh^−/−^ Cfd^−/−^ mice, in which glomerular C3 and C5b‐9 deposition was present in a mesangiocapillary distribution, which is consistent with ongoing complement activity in the kidney.^53^ Although the deposition of C5b‐9 confirms the uncontrolled activation of the alternative pathway, we cannot be sure whether it originates from a systemic deficiency of Cfh rather than from a primary defect in the regulation of retinal complement. Nonetheless, the retinal consequences of this systemic dysregulation are relevant to AMD, where systemic variants of the complement factor influence the risk of local disease.

However, key limitations must be addressed because the model does not spontaneously progress to GA or CNV, limiting its direct utility for studying late‐stage AMD. Other limitations are the absence of drusen‐like deposits similar to AMD patients and the absence of macula in mice, restricting translation of focal pathology. The absence of macula in mice would probably explain this limitation, and the environmental modulators of AMD (diet, smoking, light exposure) were not manipulated, offering avenues for future study to drive phenotype progression. Mice lack a macula, and therefore the DK model cannot recapitulate macula‐specific pathologies such as drusen topography or foveal vulnerability. Our findings are best interpreted as reflecting generalized retinal responses to complement–lipid dysregulation. Along with the absence of drusen and of macula in the animal model, methodologically, the absence of OCT images prevents direct comparison with the early stratification of AMD in humans.

Unlike single‐knockout models such as ApoE^−/−^ or Cfh^−/−^ mice, which capture only isolated aspects of AMD pathogenesis (lipid dysregulation or complement dysregulation, respectively), the DK ApoE^−/−^/Cfh^−/−^ (DK) mouse integrates both key pathways in a single model. This multifactorial approach more accurately mirrors the complex, synergistic mechanisms underlying human AMD, including RPE‐BM complex degeneration, drusen‐like deposits, complement activation (C5b‐9 deposition), dyslipidemia, chronic inflammation, and photoreceptor‐synaptic impairment. By recapitulating the structural, molecular, and functional hallmarks of early‐to‐intermediate AMD at 12 months old, the DK model fills a critical gap in AMD research: the lack of animal models that combine complement and lipid pathway deficiencies without requiring additional environmental manipulations. This creates an ideal scenario for testing inhibitors of complement system factors such as C3, C5, factor D, and factor B, whose effects can be subtle in less aggressive models. Furthermore, the DK mouse allows for the study of compensatory anti‐angiogenic responses (e.g., endostatin upregulation) alongside pro‐angiogenic signals, providing a more complete platform for testing combination therapies before advancing to retinal degeneration. Most animal models reproduce late or very aggressive stages; however, DK can better reproduce subtle changes in the RPE, alterations in BM, activated subretinal microglia, and structural alterations at a pre‐drusen stage and exhibited a progressive outer and inner retinal remodeling. This is key to therapies that aim for prevention rather than rescue.

Comparing the DK phenotype to published *ApoE* ^*−/−*^ and *Cfh* ^*−/−*^, single‐knockout models reveal both additive and potentially synergistic effects. BM thickening and lipid deposition are well described in *ApoE* ^*−/−*^ mice,^40^, ^41^ and DK mice show similar or slightly accentuated features, suggesting these are primarily driven by ApoE deficiency. C5b‐9 deposition and complement dysregulation are characteristic of *Cfh* ^*−/−*^ mice^14^ and *Cfh* ^*−/−*^ mice with CNV.^53^ DK mice exhibit comparable patterns, indicating that complement activation is largely attributable to Cfh deletion. However, the severity of photoreceptor OS disorganization and synaptic loss in DK mice exceeds that reported in either single knockout alone, suggesting a possible synergistic interaction between lipid and complement pathways in driving neuronal pathology. RPE thinning and ZO‐1 disruption were not prominently reported in single knockouts and may represent a synergistic outcome. Formal additive/synergistic assessment would require direct head‐to‐head comparison of all three genotypes (*ApoE* ^*−/−*^, *Cfh* ^*−/−*^, and DK) under identical experimental conditions, which is an important direction for future studies.

In summary, the ApoE^−/−^/Cfh^−/−^ DK mouse develops a complex retinal phenotype that exhibits concurrent features of complement dysregulation, lipid imbalance, RPE degeneration, BM remodeling, and photoreceptor‐synaptic impairment. It is worth considering that some of these changes may not be entirely specific to early–intermediate AMD as originally hypothesized. Instead, they could reflect more generalized mechanisms of retinal degeneration, common to various forms of chronic retinal stress, such as chronic inflammation, complement overactivation, and lipid homeostatic imbalance. Although the phenotype recapitulates several hallmarks associated with AMD, the possibility remains that the observed degenerative changes, particularly those affecting photoreceptor viability and synaptic integrity, represent a nonspecific response to prolonged RPE dysfunction and immune dysregulation rather than a faithful recapitulation of AMD‐specific pathogenesis. This distinction is critical, as similar features have been documented in other mouse models of retinal degeneration^51^ not primarily linked to AMD, including those related to inherited retinal dystrophies or generalized aging. Therefore, caution is warranted when attributing all observed changes exclusively to AMD‐like mechanisms, and future studies should aim to dissect which components of the DK phenotype are truly specific to AMD pathways, and future studies incorporating environmental stressors (e.g., high‐fat diet, light damage) could be considered to study the progression toward GA or CNV and the differences between aging and disease.

## AUTHOR CONTRIBUTIONS

**Sergio Recalde:** Conceptualization; investigation; methodology; visualization; writing – original draft. **Maite Moreno Orduña:** Formal analysis; methodology. **Jaione Bezunartea:** Formal analysis; methodology; software. **Idoia Belza:** Formal analysis; methodology. **Ainara Chas:** Formal analysis; methodology. **Laura Fernández‐Sánchez:** Methodology; software. **Nicolás Cuenca Navarro:** Funding acquisition; methodology; writing – review and editing. **Alfredo García‐Layana:** Conceptualization; funding acquisition; resources; supervision; validation; writing – review and editing. **Patricia Fernández‐Robredo:** Conceptualization; project administration; resources; supervision; validation; writing – review and editing. **María Hernández:** Conceptualization; data curation; investigation; methodology; supervision; writing – original draft; writing – review and editing.

## FUNDING INFORMATION

This study was funded by the Inflammatory Disease Network (RICORS‐REI; RD21/0002/0010), Red del Complemento y Salud (SAF2016‐81876‐REDT), Red del Complemento de la Comunidad de Madrid (Complemento 2‐CM), Fundación Gangoiti (Spain), and Fundación Multiópticas (Spain).

## CONFLICT OF INTEREST STATEMENT

Alfredo García‐Layana is a consultant for Bayer, Novartis, Allergan, Thea, and Roche. The rest of the authors declare no conflict of interest. The funders had no role in the design of the study; in the collection, analyses, or interpretation of data; in the writing of the manuscript; or in the decision to publish the results.

## ETHICS STATEMENT

All animal experiments were approved by the Animal Research Ethics Committee of the University of Navarra and strictly adhered to animal ethics guidelines (approval no.: 140‐12).

## Supporting information


**Table S1.** Commercial reference, dilution, and localization of the molecular markers used in the study.
