# Supplementary material for: ApoE‐ and Cfh‐deficient mice exhibit structural and molecular features of human early–intermediate retinal degeneration
Source: Animal Model Exp Med. 2026 Jul 1;9(7):1420–35. doi: 10.1002/ame2.70243 (PMC13394217; doi:10.1002/ame2.70243)
Supplement: Supplementary file 1 — Table S1. Commercial reference, dilution, and localization of the molecular markers used in the study. [file AME2-9-1420-s001.pdf]

MARKER	REFERENCE	DILUTION	LOCALIZATION
**Bassoon** mouse monoclonal	Stressgen	1:5000	photoreceptor synaptic ribbons
**C5b9** rabbit polyclonal	Biorbyt, orb499686	1:250	C5b-9, or membrane attack complex
**Calbindin** rabbit polyclonal	Swant, CB38a	1:1000	Cells in ONL and INL (cones, bipolar and amacrine cells)
**Caspase-1** rabbit monoclonal	ab207808	1:100	Apoptotic cells
**PKC-α** rabbit policlonal	Sta Cruz	1:100	Bipolar cells (INL)
**Synaptophysin** mouse monoclonal	Sigma, S5768	1:300	Synapsis (OPL and IPL)
**γ-transducin** rabbit polyclonal	Cytosignal,	1:500	Rod outer segment
**ZO-1** mouse monoclonal (Alexa Fluor™ 488)	Thermo Fisher, 339188	1:100	RPE tight junction
Donkey anti-mouse 594	Thermo Fisher, A-21203	1:250	Secondary antibody
Donkey anti-rabbit 488	Invitrogen, A21206	1:250	Secondary antibody

Table S1. Commercial reference, dilution and localization of the molecular markers used in the study.
